# Reducing New Persistent Opioid Use After Surgery: A Review of Interventions

**DOI:** 10.1007/s11916-021-00943-6

**Published:** 2021-03-24

**Authors:** Stacey Burns, Richard Urman, Rachel Pian, Oscar Jim Michael Coppes

**Affiliations:** 1 Department of Anesthesiology, Perioperative and Pain Medicine, Brigham and Women’s Hospital, 75 Francis Street, Boston, MA 02115 USA; 2 Center for Perioperative Research, Department of Anesthesiology, Perioperative and Pain Medicine, Brigham and Women’s Hospital, Harvard Medical School, Boston, MA USA; 3 Department of Biology, Boston University, Boston, MA USA; 4 Department of Anesthesia and Critical Care, University of Chicago, Chicago, IL USA

**Keywords:** Persistent postoperative opioid use, Postsurgical opioids, Multimodal analgesia, Multidisciplinary pain management, Perioperative opioid reduction

## Abstract

**Purpose of Review:**

This review aims to summarize interventions used in the perioperative period to reduce the development of new persistent postoperative opioid use in opioid-naïve patients.

**Recent Findings:**

The development of new persistent opioid use after surgery has recently been identified as a common postoperative complication. The existing literature suggests that interventions across the continuum of care have been shown to decrease the incidence of new persistent postoperative opioid use. Specific preoperative, intraoperative, and postoperative interventions will be reviewed, as well as the use of clinical pathways and protocols that span throughout the perioperative period. Common to many of these interventions include the use of multimodal analgesia throughout the perioperative period and an emphasis on a patient-centered, evidence-based approach to the perioperative pain management plan.

**Summary:**

While the incidence of new persistent postoperative opioid use appears to be high, the literature suggests that there are both small- and large-scale interventions that can be used to reduce this. Technological advances including prescription monitoring systems and mobile applications have enabled studies to monitor opioid consumption after discharge. Interventions that occur preoperatively, such as patient education and expectation setting regarding postoperative pain management, and interventions that occur postoperatively, such as the implementation of procedure-specific, evidence-based prescribing guidelines and protocols, have been shown to reduce post-discharge opioid consumption. The use of multimodal analgesia and opioid-sparing adjuncts throughout the perioperative period is central to many of these interventions and has essentially become standard of care for management of perioperative pain.

## Introduction

It is well known that the USA is facing an ongoing opioid crisis, with the number of opioid overdoses and deaths on the rise. Between 1999 and 2008 opioid prescribing in the USA quadrupled, with a parallel increase in reported overdoses [1]. Approximately 40% of opioid overdose deaths involve a prescription opioid, with the combined economic burden of prescription opioid-related overdose, abuse, and dependence exceeding $78.5 billion annually [2]. Given this grave reality alongside the multitude of well-documented adverse effects of opioids, interventions have been implemented across all levels of the healthcare system with the aim of limiting opioid prescriptions.

Surgical patients are particularly vulnerable during the perioperative period, as the majority of them are exposed to opioids at some point during this time. Up to 14.7% of patients who are opioid-naïve may develop persistent postoperative opioid use [3], and studies show that opioids prescribed during and after surgery can increase the risk of chronic use in all patients [4–7]. Additionally, the incidence of chronic postsurgical pain can be as high as 85% depending on the type of surgery [7]. New persistent opioid use after surgery, most commonly defined as continued opioid prescription use between 90 and 180 days after the surgical procedure in a previously opioid-naïve patient, has recently been identified as one of the most common postoperative complications [3, 8]. Therefore, this population has become an increasingly important focus in the literature.

The incidence of new persistent opioid use after surgery is multifactorial, related to patient-specific risk factors, the type of surgery, and prescribing behaviors during the perioperative period. While there have been various interventions designed to decrease opioid use in the immediate perioperative period, lack of long-term longitudinal follow-up precludes an accurate assessment of the impact on new postoperative opioid use. This article will discuss risk factors associated with persistent opioid use after surgery and provide an up-to-date review of interventions designed to prevent new persistent opioid use after surgery. Interventions under discussion will include perioperative protocols/clinical pathways and programs, as well as preoperative-, intraoperative-, and postoperative-specific interventions.

## Risk Factors for the Development of New Persistent Postoperative Opioid Use

Risk factors associated with the development of persistent postoperative opioid use in previously opioid-naïve patients include patient-related risk factors, surgical factors, and the perioperative pain management plan. Multiple patient-specific variables have been identified as potentially contributing to new persistent opioid use, including substance use disorders, mood disorders, and preexisting chronic pain [6, 9••]. Specific surgical procedures that have been linked to an increased risk of persistent postoperative opioid use include total knee arthroplasty, simple mastectomy, open cholecystectomy, open appendectomy, cesarean delivery, and cardiothoracic surgery [6, 10]. Additionally, many studies have shown that the amount and duration of opioids prescribed at discharge contribute to the development of new persistent postoperative opioid use [4, 5, 10, 11••]. Knowledge of these risk factors can aid in the early identification of patients at high risk, which may significantly alter a patient’s trajectory of care.

## Perioperative Protocols/Clinical Pathways

The implementation of multidisciplinary perioperative protocols which focus on the use of opioid-sparing multimodal analgesia modalities throughout the continuum of care, like Enhanced Recovery after Surgery (ERAS) protocols, have consistently shown to be associated with decreased perioperative opioid consumption. While the majority of studies examining the effects of ERAS protocols on perioperative opioid use terminate at patient discharge, tracking post-discharge opioid use has enabled more recent studies to examine the impact of ERAS protocols on both discharge and post-discharge opioid prescriptions. Liu et al. (2019) implemented an ERAS protocol targeting two patient populations (2468 elective colorectal and 3885 emergent hip fracture patients) with specific multimodal non-opioid adjuncts and followed patients for up to 1 year after surgery. Using pharmacy dispensing records it was reported that implementation of the ERAS protocol was associated with more than a 20% reduction in opioid refills 3 months to 1 year after surgery [12]. Razi et al. (2020) investigated an Enhanced Recovery after Thoracic Surgery (ERATS) protocol for patients undergoing thoracotomies and robotic thoracoscopies which focused on a multimodal pain management strategy including the use of opioid-sparing analgesics and regional anesthesia. Implementation of the ERATS protocol resulted in a drastic reduction of new persistent opioid use without adversely affecting outcomes [13]. Similar longitudinal results have been reported in liver surgery [14], indicating potentially unrealized benefits of ERAS protocols that extend beyond discharge.

Technological advances have enabled the incorporation of novel mobile applications into ERAS protocols, which can aid in tracking postoperative opioid use. Pickens et al. (2019) found that the implementation of a mobile application for real-time collection of patient-reported outcomes for 30 days after hepatopancreatobiliary surgery within an ERAS pathway resulted in decreased opioid consumption and pain scores, increased compliance with ambulation and breathing exercises, and prevention of emergency room visits [15]. Future adoption and scaling of such mobile applications may prove to be beneficial in the management and tracking of postsurgical pain and opioid use.

Another example of a multidisciplinary, integrative approach to the prevention of new persistent postoperative opioid use is Toronto General Hospital’s Transitional Pain Service, which is designed to identify patients at risk for chronic postsurgical pain and opioid use and offer coordinated and comprehensive care throughout the perioperative period. Patients are seen preoperatively, postoperatively in the hospital, and after discharge in an outpatient setting for up to 6 months after surgery [16]. Clarke et al. (2018) found that participation in the Transitional Pain Service significantly improved pain scores and simultaneously reduced the incidence of persistent opioid use within the first 6 months after surgery [17].

## Preoperative Interventions

The preoperative period is a critical stage where patients with risk factors for the development of new persistent opioid use can be identified early on, which can prompt the early involvement of programs like Toronto General Hospital’s Transitional Pain Service. In these patients, it is important to obtain baseline pain levels prior to surgery, as this information can help to prevent overzealous treatment of postoperative pain [8]. Studies across various surgical subspecialties have highlighted the importance of preoperative-specific interventions on reducing new persistent opioid use after surgery. Overall, interventions emphasizing patient education, expectation setting, shared decision making, and early identification of high-risk patients are associated with a decrease in the incidence of new persistent postoperative opioid use [18–22]. A recent systematic review of institutional strategies aimed at reducing opioids after orthopedic surgery reported that formal preoperative patient education programs were the most effective intervention in reducing persistent postoperative opioid use [18]. In patients undergoing hysterectomies, those who participated in a preoperative patient education program consumed significantly fewer oral morphine equivalents at the time of discharge with no significant change in patient satisfaction or number of refills. This education program included a discussion of pain expectations and information regarding the mean number of opioids used by a similar cohort, and patients were then able to choose the number of opioids prescribed at discharge [19]. Similarly, a recently published randomized controlled trial examining the effect of opioid-related preoperative patient education on post-discharge opioid consumption in patients undergoing arthroscopic rotator cuff repair reported that preoperative patient education significantly decreased the number of opioid pills consumed at 3 months after surgery. Furthermore, it led to earlier cessation of opioids in both opioid-naïve and opioid-tolerant patients [23].

## Intraoperative and Immediate Perioperative Interventions

Over the past 20 years, there has been a paradigm shift in the management of perioperative pain, with an evolving focus on the use of multimodal analgesia in the immediate perioperative period to reduce unnecessary opioid exposure. Multimodal analgesia, referred to above, is defined as two or more interventions with varying mechanisms of action for postoperative pain relief [24]. It has been suggested that by increasing the number of non-opioid pain management modalities, more effective pain control is achieved with decreased opioid use [25]. Examples of opioid-sparing multimodal interventions include pharmacological adjuncts like ketamine, nonsteroidal anti-inflammatory drugs, acetaminophen, gabapentinoids, intravenous lidocaine infusions, regional and neuraxial techniques, and non-pharmacologic techniques such as TENS, acupuncture, and music therapy [26]. Liposomal bupivacaine is newly being considered, although data is still developing. The use of multimodal adjuncts in the immediate perioperative period has been studied extensively [10, 26, 27••], and while many of these adjuncts have been shown to be associated with reduced opioid consumption and pain scores in the immediate postoperative period, most studies do not continue to track opioid use after discharge.

## Postoperative Interventions

Given the lack of large-scale data on post-discharge pain trajectories and opioid usage, lack of guidance regarding optimal postoperative pain management, and the resulting widespread variability in prescribing practices, multiple studies have focused on postoperative-specific interventions to decrease opioid consumption after discharge [24]. Many surgical specialties are now implementing evidence-based, procedure-specific postoperative pain management protocols to help guide opioid prescribing practices, resulting in decreased variability and decreased unnecessary opioid prescriptions [20, 28–30].

Provider education and implementing procedure-specific prescribing guidelines for discharge have consistently been shown to reduce new persistent opioid use [18, 31–34]. In a study by Kaafarni et al. (2019), persistent postoperative opioid use was decreased after implementation of a multidisciplinary intervention including opioid guidelines for procedures from 11 different specialties, provider-focused posters displayed in all surgical units, patient opioid/pain brochures setting expectations, and educational seminars to residents, advanced practice providers, and nurses [35]. Similarly, a 2020 study by Patel et al. found that implementing an opioid reduction intervention consisting of an informational sheet for patients at discharge, education by nurses at discharge, and an evidence-based prescribing guideline resulted in decreased opioid usage in the first 30 days after surgery [36]. Interestingly, Howard et al. (2018) found that after implementing evidence-based opioid prescribing recommendations for a single surgical procedure (laparoscopic cholecystectomy), discharge opioid prescriptions decreased for four other unrelated surgical procedures without significantly affecting refill rates, described as the “spillover effect” [37]. In addition to procedure-specific protocols, recent studies suggest that patient-specific prescribing and tapering protocols based on a review of the patient’s personal opioid consumption prior to discharge leads to decreased persistent opioid use after discharge [38, 39]. Other simple yet effective interventions include lowering discharge opioid prescription defaults in the electronic health system [40] and the creation of evidence-based procedure-specific discharge ordering sets [41]. Involvement of a transitional pain service can help to bridge the potential gap in care after discharge.

Lastly, a review of a novel electronic health modality aimed at optimizing pain management while decreasing unnecessary opioid consumption was recently published, in which the authors designed and implemented a mobile application guiding patients in pain management and opiate use in the first 2 weeks after discharge from a total knee replacement. They reported that use of the application resulted in decreased opioid consumption, and more frequent use was associated with a more pronounced result [42••]. While this study is limited in the duration of follow-up, further technological advances will continue to pave the way for closer monitoring of patients after surgical discharge, enabling more tailored, collaborative patient-centered care.

## Conclusions

With growing evidence that new persistent opioid use after major and minor surgery is relatively common, there has been an increased interest in longitudinal follow-up of postoperative opioid use. Efforts are being made across the continuum of care to limit unnecessary exposure to opioids. Current literature suggests that interventions can be performed throughout the perioperative period to successfully decrease the risk of patients developing new persistent postoperative opioid use. Early identification of patients at risk can help to tailor interventions within the context of a multidisciplinary perioperative pain management plan. The use of multimodal anesthesia and analgesia, enhanced recovery pathways, and additional opioid-sparing techniques have become the standard of care in the management of perioperative pain. While many studies examining single interventions in isolation have shown to consistently decrease opioid requirements in the immediate perioperative period, few studies report the impact on opioid consumption after discharge and the same is true for many of the ERAS studies. Future studies are needed to determine if the observed decrease in in-hospital opioid use translates into decreased post-discharge opioid use and the development of new persistent use. Fortunately, technological advances including prescription monitoring and centralized electronic medical record systems are making post-discharge tracking more feasible, which will enable future studies the ability to follow patients in the longer term. Additionally, the use of mobile electronic applications as a way to decrease post-discharge opioid consumption appears to be a promising new development, enabling closer follow-up, monitoring, and patient-centered prescription guidance.
